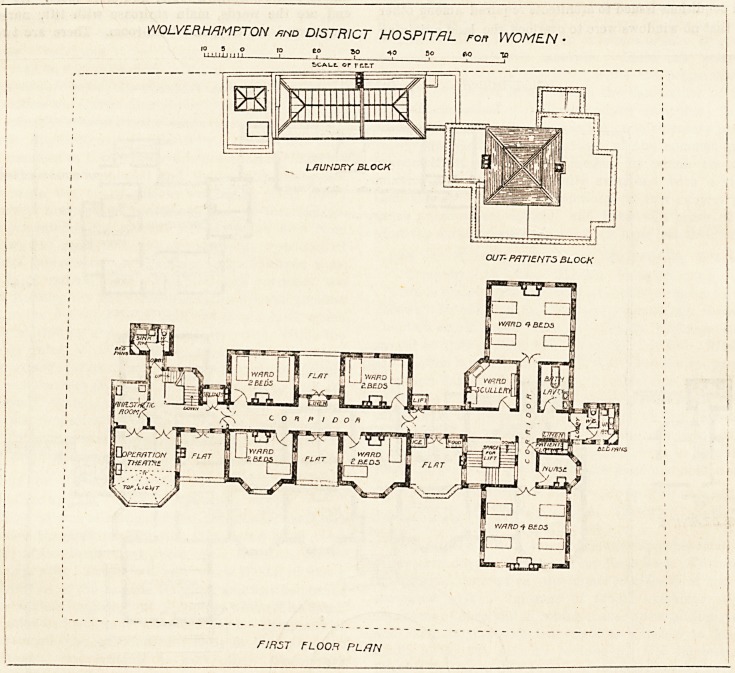# Wolverhampton Hospital for Women

**Published:** 1904-10-29

**Authors:** 


### HOSPITAL ADMINISTRATION.

### CONSTRUCTION AND ECONOMICS.

### WOLVERHAMPTON HOSPITAL FOR WOMEN.

The site for this hospital was a generous gift by Mr. Gibbons, of Ellowes Hall, Staffordshire, who is chairman of the committee of management. The main frontage of the building is towards Park Road West, so that the hospital looks over the prettily laid out West End Park, and the special conditions issued to architects required among other things that no windows were to overlook the land adjoining the south-east boundaries of the site, and that the kitchens were to be placed in the basement. Only local architects were allowed to compete, and the assessor was Mr. T. W. Aldwinkle. The successful competitor was Mr. Eaton Painter, of Wolverhampton, and we may say at once that his plan is a very creditable production, and, taking into consideration its size and purpose, one of the best designs we have yet published in The Hospital. The cost was £11,400, and the work was carried out by Mr. Gough.

The ground floor of the main building is about 5 feet above the level of the site, and is approached by a semi-circular drive, from the centre of which the entrance is reached by a flight of steps. The block is divided longitudinally by a corridor, and crossways by the entrance hall and waiting-lobby. On the right to the north are the nurses' sitting-room and dining-room, and on the left are the matron's room and board-room. On the south of the corridor are nurses' bedrooms, linen-room, and store-room. At the west end are the wards, main staircase with lift, nurses' duty room, ward scullery, and bath-room. There are two wards for four beds each, one being to the south and the other to the north. The rooms are well lighted and cross-ventilated, and each bed has a window on both sides of it. The first floor has two wards of the same size, and all the adjuncts have the same satisfactory arrangement as on the ground floor. There are also four two-bedded rooms on the first floor, and as there are no rooms over the hall, waiting-room, nurses' dining-room, and matron's bedroom, very good cross ventilation is obtained for these two-bedded rooms. This point is so often overlooked by architects when designing single or double bedded rooms, that the simple and efficient means by which Mr. Painter has obtained the essential cross current deserve notice. The first floor rooms named as being not carried up, have lead flat roofs: hence in case of fire they could be used as escapes, and they will afford a pleasant place in which patients may sit, or on which they may be wheeled in their bedsteads to get sunshine and air.

The sanitary blocks are well cut off from the main by cross-ventilated passages; in fact, these passages are really covered bridges. It seems almost a pity that the bath-room could not have been placed in the annexe, as it is certainly desirable to have it also cut off from the main. At the same time it may be admitted that in so small a hospital this drawback will not be seriously felt, and we notice that the

WOLVERHAMPTON AND DISTRICT HOSPITAL FOR WOMEN.

FIRST FLOOR PLAN

ground floor bath has been placed angle-ways in the room so that it can be approached from all sides.

The operating-room is placed on the second floor and over the board-room. It has abundance of roof light, and faces the north. An anæsthetic-room adjoins it.

The top floor of the main building contains bedrooms for the night nurses and for the domestic servants.

The smaller block includes the out-patients' department and the laundry. The former contains a large central waiting-hall, and round it are placed the medical officers' consulting-room, the dressing-rooms, dispensary, and medicine waiting-room. The out-patients' department has a separate entrance from Connaught Road.

The laundry department is conveniently arranged.

The mortuary is placed at the north-east end.

## Figures and Tables

**Figure f1:**
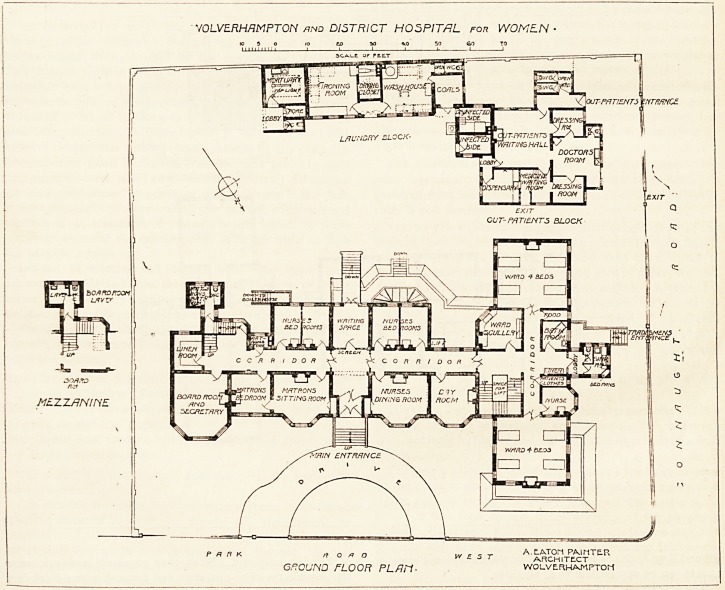


**Figure f2:**